# Distribution of Link Distances in a Wireless Network

**DOI:** 10.6028/jres.106.017

**Published:** 2001-04-01

**Authors:** Leonard E. Miller

**Affiliations:** National Institute of Standards and Technology, Gaithersburg, MD 20899-8920

**Keywords:** link distance, mobile networks, probability distribution, wireless communication, wireless networks

## Abstract

The probability distribution is found for the link distance between two randomly positioned mobile radios in a wireless network for two representative deployment scenarios: (1) the mobile locations are uniformly distributed over a rectangular area and (2) the *x* and *y* coordinates of the mobile locations have Gaussian distributions. It is shown that the shapes of the link distance distributions for these scenarios are very similar when the width of the rectangular area in the first scenario is taken to be about three times the standard deviation of the location distribution in the second scenario. Thus the choice of mobile location distribution is not critical, but can be selected for the convenience of other aspects of the analysis or simulation of the mobile system.

## 1. Introduction

The probability that a link between two mobile radios has sufficient signal-to-noise ratio for acceptable transmission quality or reliability is, other factors being equal, the probability that the link distance *d* is less that some value *R*, where *R* is termed the transmission range:

$$\mathrm{Pr}\left\{\text{Link}\;\text{is}\;\text{good}\right\}=\mathrm{Pr}\left\{d\leq R\right\}=F_{d}\left(R\right).$$ (1.1)

The function F*_d_*(·) in Eq. (1.1) is the cumulative probability distribution function (cdf) for the link distance.

Assuming that different links fail independently, the quantity F*_d_*(*R*) can be taken as the probability of success (acceptable transmission quality) in a binomial trial in which two link endpoints are selected; if the trial is repeated *N* times, then an estimate of the number of good links is *N*F*_d_*(*R*). Also, the probability that multihop communication paths are reliable can be related to the individual link reliabilities. For these and other reasons, the cdf for the link distances in a mobile radio system is an important quantity [1,2].

There is an infinite number of potential scenarios in which locations are selected for the different mobile radios. In this paper, in order to lay the goundwork for further analysis of mobile radio systems, a random selection of mobile locations is assumed, and the cdf of the link distances is found for two simple but fundamental scenarios: (1) a rectangular deployment area in which mobiles are uniformly distributed and (2) a deployment in which the *x* and *y* coordinates of the mobile locations have Gaussian distributions.

## 2. Uniform Distribution of Link Distances in a Rectangular Area

### 2.1 Assumptions and Formulation of the Derivation

**Fig. 1:**
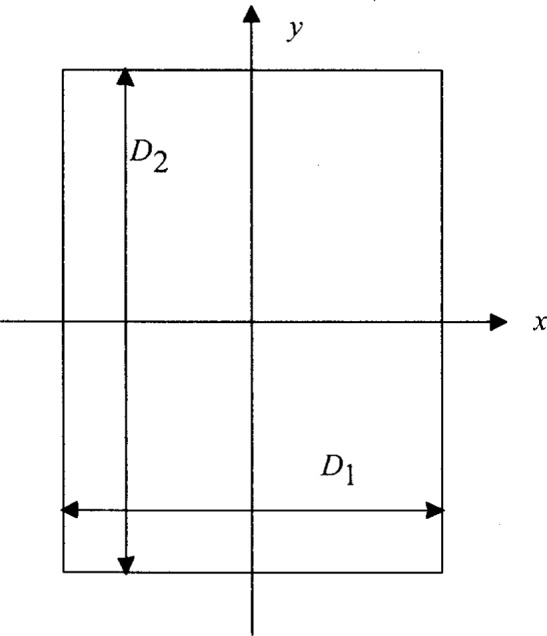
Rectangular area for uniform distribution of mobile locations.

Let the positions of the mobile users (referred to as “mobiles”) be distributed randomly in a rectangular area with dimensions *D*_1_ and *D*_2_, as illustrated in Fig. 1, in which we have assumed *D*_1_ ≤ *D*_2_ without loss of generality. The *x_i_* and *y_i_* coordinates of mobile *i* have the uniform distributions given by the probability density functions (pdfs) *p_x_*(*α*) and *p_y_*(*β*), respectively, where

$$p_{x}\left(\alpha \right)=\{\begin{array}{ll} \frac{1}{D_{1}},\; & \left|\alpha \right|\leq \frac{1}{2}D_{1}\\ 0, & \text{otherwise} \end{array}$$ (2.1a)

$$p_{y}\left(\beta \right)=\{\begin{array}{ll} \frac{1}{D_{2}},\; & \left|\beta \right|\leq \frac{1}{2}D_{2}\\ 0, & \text{otherwise} \end{array}.$$ (2.1b)

We assume that the *x* and *y* positions of any two mobiles are selected independently.

**Fig. 2:**
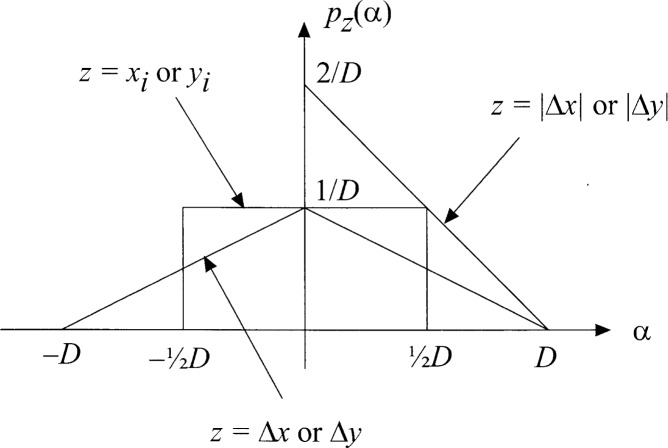
Pdfs for location, difference, and absolute value of difference.

The link distance between mobiles *i* and *j* is defined as

$$d_{ij}≜\sqrt{{\left(x_{i}-x_{j}\right)}^{2}+{\left(y_{i}-y_{j}\right)}^{2}}=\sqrt{{\left(\Delta x\right)}^{2}+{\left(\Delta y\right)}^{2}}$$ (2.2)

where, as illustrated generically in Fig. 2, the differences Δ*x* = *x_i_ − x_j_* and *y* = *y_i_* − *y_j_* are independent and have the pdfs given by

$$p_{\Delta x}\left(\alpha \right)=\{\begin{array}{ll} \frac{D_{1}-\left|\alpha \right|}{D_{1}^{2}}, & \left|\alpha \right|\leq D_{1}\\ 0, & \text{otherwise} \end{array}$$ (2.3a)

and

$$p_{\Delta y}\left(\beta \right)=\{\begin{array}{ll} \frac{D_{2}-\left|\beta \right|}{D_{2}^{2}}, & \left|\beta \right|\leq D_{2}\\ 0, & \text{otherwise} \end{array}$$ (2.3b)

and where the absolute values of the differences |Δ*x*| = |*x_i_* − *x_j_*| and |Δ*y*| = |*y_i_* − *y_j_*| are independent and have the pdfs given by

$$p_{\left|\Delta x\right|}\left(\alpha \right)=\{\begin{array}{ll} \frac{2\left(D_{1}-\alpha \right)}{D_{1}^{2}}, & 0\leq \alpha \leq D_{1}\\ 0, & \text{otherwise} \end{array}.$$ (2.4a)

and

$$p_{\left|\Delta y\right|}\left(\beta \right)=\{\begin{array}{ll} \frac{2\left(D_{2}-\beta \right)}{D_{2}^{2}}, & 0\leq \beta \leq D_{2}\\ 0, & \text{otherwise} \end{array}.$$ (2.4b)

**Fig. 3:**
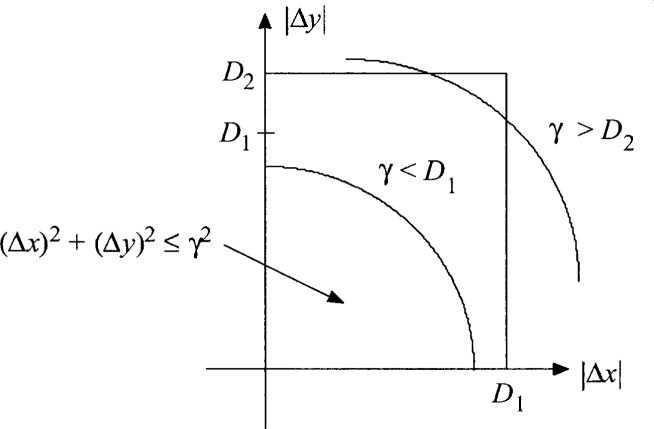
Domain of integration for the pdf.

The cumulative probability distribution function for the distance between two mobiles therefore is formulated as

$$\begin{array}{l} F_{d}\left(\gamma \right)=\mathrm{Pr}\left\{d_{ij}\leq \gamma \right\}=\mathrm{Pr}\left\{\surd \bar{{\left(x_{i}-x_{j}\right)}^{2}+{\left(y_{i}-y_{j}\right)}^{2}}\leq \gamma \right\}\\ =\mathrm{Pr}\left\{\sqrt{{\left(\Delta x\right)}^{2}+{\left(\Delta y\right)}^{2}}\leq \gamma \right\}=\mathrm{Pr}\left\{\sqrt{{\left|\Delta x\right|}^{2}+{\left|\Delta y\right|}^{2}}\leq \gamma \right\}\\ =\iint_{\left(\alpha ,\beta \right)\in A}d\alpha \;d\beta p_{\left|\Delta x\right|,\left|\Delta y\right|}\left(\alpha ,\beta \right) \end{array}$$ (2.5a)

$$=1-\iint_{\left(\alpha ,\beta \right)\notin A}d\alpha \;d\beta p_{\left|\Delta x\right|,\left|\Delta y\right|}\left(\alpha ,\beta \right)$$ (2.5b)

where *p*_|Δ_*_x_*_|,|Δ_*_y_*_|_(*α*, *β*) = *p*_|Δ_*_x_*_|_ (*α*)*p*_|Δ_*_y_*_|_(*β*) denotes the joint pdf of the absolute values of the *x* and *y* differences and *A* denotes the domain of integration, illustrated in Fig. 3, such that $\sqrt{\alpha^{2}+\beta^{2}}\leq \gamma$ while both 0 ≤ *α* ≤ *D*_1_ and 0 ≤ *β* ≤ *D*_2_, or 0 ≤ *α* ≤ min{*D*_1_, *γ*} and $0\leq \beta \leq \min\left\{D_{2},\sqrt{\gamma^{2}-\alpha^{2}}\right\}$. Using the pdfs of Eqs. (2.4a) and (2.4b), Eq. (2.5a) becomes

$$\begin{array}{c} F_{d}\left(\gamma \right)=\int_{0}^{\min\left\{D_{1},\gamma \right\}}d\alpha \\ \int_{0}^{\min\left\{D_{2},\sqrt{\gamma^{2}-\alpha^{2}}\right\}}d\beta \frac{4}{D_{1}D_{2}}\left(1-\frac{\alpha }{D_{1}}\right)\left(1-\frac{\beta }{D_{2}}\right) \end{array}$$ (2.6a)

$$=4\int_{0}^{\min\left\{1,\gamma /D_{1}\right\}}du\left(1-u\right)\int_{0}^{\min\left\{1,\sqrt{\gamma^{2}-D_{1}^{2}u^{2}}/D_{2}\right\}}dv\left(1-v\right)$$ (2.6b)

$$=4\int_{0}^{\min\left\{1,\xi \right\}}du\left(1-u\right)\int_{0}^{\min\left\{1,\zeta \sqrt{\xi^{2}-u^{2}}\right\}}dv\left(1-v\right)$$ (2.6c)

in which we define the normalized variable *ξ* ≜ *γ*/*D*_1_ and the area shape parameter *ζ* ≜*D*_1_*D*_2_ ≤ 1. The evaluation of this double integral is facilitated by considering different intervals for the value of *γ*. For *γ* < 0, of course, the integral equals zero. For $\gamma >\sqrt{D_{1}^{2}+D_{2}^{2}}$, the double integral equals one. Similarly, Eq. (2.5b) becomes

$$F_{d}\left(\gamma \right)=1-\int_{L_{1}}^{D_{1}}d\alpha \int_{L_{2}\left(\alpha \right)}^{D_{2}}d\beta \frac{4}{D_{1}D_{2}}\left(1-\frac{\alpha }{D_{1}}\right)\left(1-\frac{\beta }{D_{2}}\right)$$ (2.7a)

$$=1-4\int_{{L^{\prime }}_{1}}^{1}du\left(1-u\right)\int_{{L^{\prime }}_{2}\left(u\right)}^{1}dv\left(1-v\right)$$ (2.7b)

with the lower limits

$$\begin{array}{l} L_{1}=\{\begin{array}{rr} 0, & 0<\gamma \leq D_{2}\\ \sqrt{\gamma^{2}-D_{2}^{2}}, & D_{2}<\gamma \leq \sqrt{D_{1}^{2}+D_{2}^{2}} \end{array}\\ {L^{\prime }}_{1}=\{\begin{array}{rr} 0, & 0<\xi \leq \zeta^{-1}\\ \sqrt{\xi^{2}-\zeta^{-2}}, & \zeta^{-1}<\xi \leq \sqrt{1+\zeta^{-2}} \end{array} \end{array}$$ (2.7c)

$$\begin{array}{l} L_{2}=\{\begin{array}{rr} 0, & 0<\gamma \leq D_{1}\;\text{and}\;\alpha >\gamma \\ \sqrt{\gamma^{2}-\alpha^{2}}, & D_{1}<\gamma \leq \sqrt{D_{1}^{2}+D_{2}^{2}} \end{array}\\ {L^{\prime }}_{2}=\{\begin{array}{rr} 0, & 0<\xi \leq 1\;\text{and}\;u>\xi \\ \zeta \sqrt{\xi^{2}-u^{2}}, & 1<\xi \leq \sqrt{1+\zeta^{-2}} \end{array}. \end{array}$$ (2.7d)

### 2.2 Representative Results for the cdf

In Appendix A, it is shown that the cdf for the link distance between two mobiles that are randomly positioned in a rectangular area is given by Eq. (2.8). For a square area with *D*_1_ = *D*_2_ = *D*, or *ζ* = 1, the cdf reduces to Eq. (2.9).

$$F_{d}\left(\gamma =\xi D_{1}\right)=\{\begin{array}{rr} 0, & \xi <0\\ \zeta \xi^{2}\left[\frac{1}{2}\zeta \xi^{2}-\frac{4}{3}\xi \left(1+\zeta \right)+\pi ,\right] & 0\leq \xi <1\\ \frac{2}{3}\zeta \sqrt{\xi^{2}-1}\left(2\xi^{2}+1\right)-\frac{1}{6}\zeta \left(8\xi^{3}+6\zeta \xi^{2}-\zeta \right) & \\ +2\zeta \xi^{2}\sin^{-1}\left(1/\xi \right), & 1\leq \xi <\zeta^{-1}\\ \frac{2}{3}\zeta \sqrt{\xi^{2}-1}\left(2\xi^{2}+1\right)-\frac{1}{2}\zeta^{2}\left(\xi^{4}+2\xi^{2}-\frac{1}{3}\right) & \\ +\frac{2}{3}\sqrt{\xi^{2}-\zeta^{-2}}\left(2\zeta^{2}\xi^{2}+1\right)+\frac{1}{6}\zeta^{-2}-\xi^{2} & \\ +2\zeta \xi^{2}\left\{\sin^{-1}\left(1/\xi \right)-\cos^{-1}\left(1/\zeta \xi \right)\right\}, & \zeta^{-1}\leq \xi <\sqrt{1+\zeta^{-2}}\\ 1, & \sqrt{1+\zeta^{-2}}\leq \xi \end{array}.$$ (2.8)

$$F_{d}(\gamma =\xi D)=\{\begin{array}{rr} 0, & \xi <0\\ \xi^{2}(\frac{1}{2}\xi^{2}-\frac{8}{3}\xi +\pi ), & 0\leq \xi <1\\ \frac{4}{3}\sqrt{\xi^{2}-1}(2\xi^{2}+1)-(\frac{1}{2}\xi^{4}+2\xi^{2}-\frac{1}{3}) & \\ +2\xi^{2}[\sin^{-1}(1/\xi )-\cos^{-1}(1/\xi )], & 1\leq \xi <\sqrt{2}\\ 1, & \sqrt{2}\leq \xi \end{array}.$$ (2.9)

**Fig. 4:**
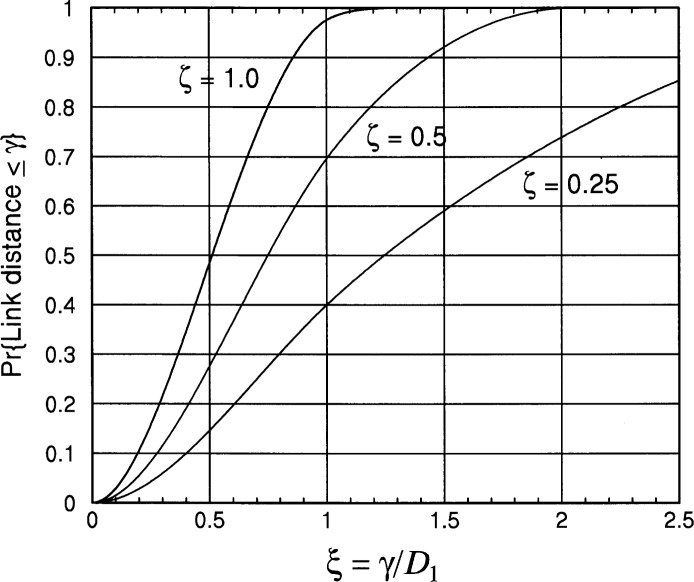
Plot of the link distance cdf for a rectangular deployment area (*D*_1_ = *ζD*_2_ ≤ *D*_2_).

Example plots of Eqs. (2.8) and (2.9) are shown in Fig. 4. For example, note from Fig. 4 that the median link distance (the value *γ* of for which the cdf equals 0.5) is approximately $\gamma =d_{\text{med}}\approx \frac{1}{2}D$ for the case of *ζ* = 1. In fact, solving F*_d_*(*ξ*) = 0.5 numerically for *ζ* = 1 yields *ξ*_med_ = 0.5120. Additional median values for this distribution are given in Table 1 for different values of *ζ*.

### 2.3 The pdf and Mode for the Link Distance in a Rectangular Area

**Fig. 5:**
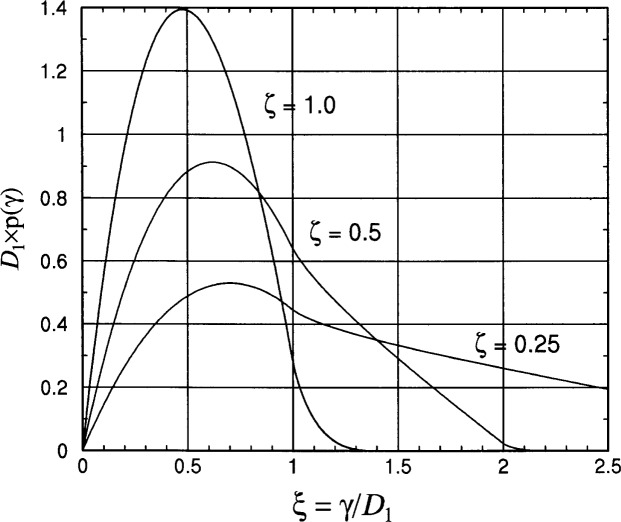
Plot of the link distance pdf for a rectangular deployment area (*D*_1_ = *ζD*_2_ ≤ *D*_2_).

The probability density function p*_d_*(*γ* = *ξ D*_1_) for the link distance in a rectangular area is found by differentiating the cdf in Eq. (2.8) to obtain Eq. (2.10). For the special case of *D*_1_ = *D*_2_ = *D* or *ζ* = 1, Eq. (2.10) becomes Eq. (2.11). Example plots of these functions are shown in Fig. 5.

$$p_{d}\left(\gamma =\xi D_{1}\right)=\frac{1}{D_{1}}\{\begin{array}{ll} \zeta \xi \left[2\zeta \xi^{2}-4\xi \left(1+\zeta \right)+2\pi \right], & 0\leq \xi <1\\ 4\zeta \xi \sqrt{\xi^{2}-1}-2\zeta \xi \left(2\xi +\zeta \right) & \\ +4\zeta \xi \sin^{-1}\left(1/\xi \right), & 1\leq \xi <\zeta^{-1}\\ 4\zeta \xi \sqrt{\xi^{2}-1}+4\zeta^{2}\xi \sqrt{\xi^{2}-\zeta^{-2}} & \\ -2\xi \left(\zeta^{2}\xi^{2}+1+\zeta^{2}\right) & \\ +4\zeta \xi \left\{\sin^{-1}\left(1/\xi \right)-\cos^{-1}\left(1/\zeta \xi \right)\right\}, & \zeta^{-1}\leq \xi <\sqrt{1+\zeta^{-2}}\\ 0, & \text{otherwise} \end{array}.$$ (2.10)

$$p_{d}\left(\gamma =\xi D\right)=\frac{1}{D}\{\begin{array}{ll} 2\xi \left(\xi^{2}-4\xi +\pi \right), & 0\leq \xi <1\\ 8\xi \sqrt{\xi^{2}-1}-2\xi \left(\xi^{2}+2\right) & \\ +4\xi \left\{\sin^{-1}\left(1/\xi \right)-\cos^{-1}\left(1/\xi \right)\right\}, & 1\leq \xi <\sqrt{2}\\ 0, & \text{otherwise} \end{array}.$$ (2.11)

From differentiation of the pdf and solving the resulting quadratic equation, the mode of the distribution is found to be

$$\xi_{\mathrm{mode}}=\frac{\gamma_{\mathrm{mode}}}{D_{1}}=\frac{2\left(1+\zeta \right)}{3\zeta }-\sqrt{\frac{4{\left(1+\zeta \right)}^{2}}{9\zeta^{2}}-\frac{\pi }{3\zeta }}.$$ (2.12)

Example values of the mode for different values of *ζ* are given in Table 2. The mode values in Table 2 are smaller than the median values in Table 1, indicating a significant amount of skew in the distribution, which can be observed in the pdf plots in Fig. 5.

## 3. Distribution of Link Distances for Gaussian-Distributed Coordinates

### 3.1 Derivation of the Link Distance pdf and cdf for Gaussian-Distributed Locations

Instead of assuming that the mobiles are randomly located in a rectangular area, we now assume that the *x* and *y* coordinates of the mobile locations have Gaussian distributions. That is, we assume that the pdfs of the *x* and *y* coordinates are independent and have the following pdfs:

$$p_{x}\left(\alpha \right)=\frac{1}{\sigma_{1}\sqrt{2\pi }}e^{-\alpha^{2}/2\sigma_{1}^{2}},\;-\infty <\alpha <\infty$$ (3.1a)

and

$$p_{y}\left(\beta \right)=\frac{1}{\sigma_{2}\sqrt{2\pi }}e^{-\beta^{2}/2\sigma_{2}^{2}},\;-\infty <\beta <\infty$$ (3.1b)

where *σ*_1_ and *σ*_2_ are, respectively, the standard deviations of the *x* and *y* coordinates. Without loss of generality, we assume that *σ*_1_ = *λσ*_2_, where *λ* is an area shape parameter, with *λ* ≤ 1. The joint pdf of the coordinates is given by

$$p_{x,y}\left(\alpha ,\beta \right)=\frac{1}{2\pi \sigma_{1}\sigma_{2}}\exp\left\{-\frac{1}{2}\left[{\left(\frac{\alpha }{\sigma_{1}}\right)}^{2}+{\left(\frac{\beta }{\sigma_{2}}\right)}^{2}\right]\right\}.$$ (3.2a)

**Fig. 6:**
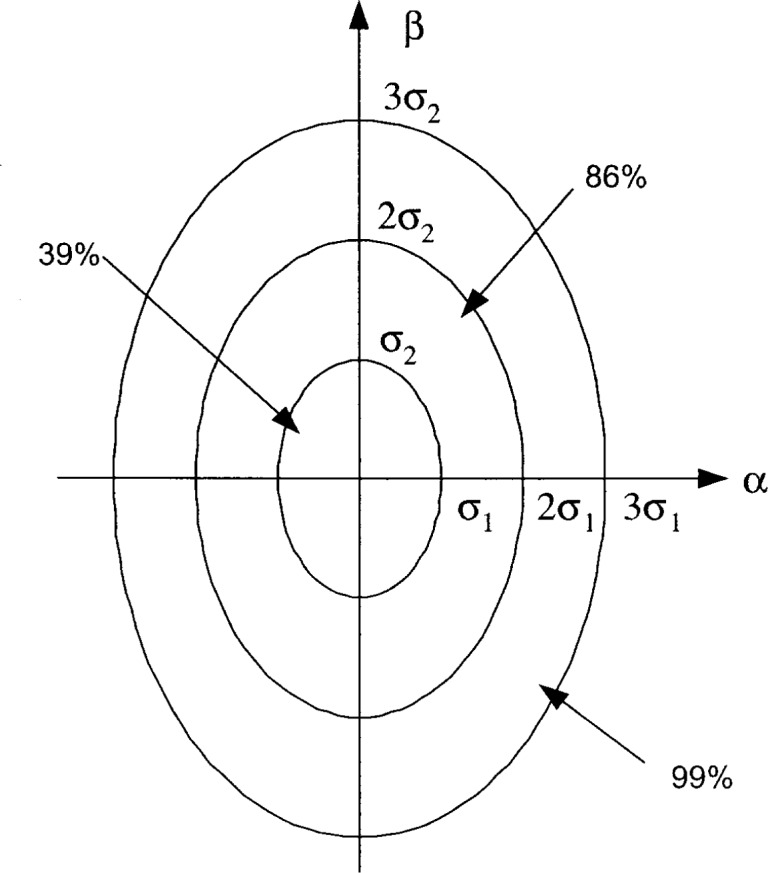
Elliptical areas associated Gaussian mobile coordinates.

Note that the joint pdf in Eq. (3.2a) is the special case of the bivariate Gaussian pdf with uncorrelated random variables (RVs); the more general case of correlated Gaussian coordinates can be treated by using a simple transformation of the coordinate system. As illustrated in Fig. 6, the elliptical area defined by the equation

$${\left(\frac{\alpha }{\sigma_{1}}\right)}^{2}+{\left(\frac{\beta }{\sigma_{2}}\right)}^{2}=k^{2}$$ (3.2b)

contains $100\left(1-e^{-k^{2}/2}\right)$ percent of the mobile positions, or about 39 % of the mobile positions when *k* = 1, 86 % when *k* = 2, and 99 % when *k* = 3. The elliptical area containing nearly all the positions corresponds to the rectangular area shown in Fig. 1, so that the Gaussian-coordinate model can easily be related to the uniformly distributed mobile model when it is convenient. For example, an ellipse just fitting inside the rectangle of Fig. 1 has the area $\frac{1}{4}\pi D_{1}D_{2}$ and contains $\frac{1}{4}\pi =78.54\%$ of the mobile positions for the rectangular, uniform distribution. This same percentage for the Gaussian-coordinate model is contained in the elliptical area given by Eq. (3.2b) with *k* = 1.754, so that the two models are roughly equivalent when $\frac{1}{2}D_{1}\approx 1.75\;\sigma_{1}$ and $\frac{1}{2}D_{2}\approx 1.75\;\sigma_{2}$ or *D*_1_ ≈ 3.5 *σ*_1_ and *D*_2_ ≈ 3.5 *σ*_2_.

Since a difference of independent Gaussian RVs with variances *a* and *b* is also a Gaussian RV whose variance is *a* + *b*, the differences in the coordinates of two mobiles are Gaussian:

$$\begin{array}{l} \Delta \;x=x_{i}-x_{j}=G\left(0,2\sigma_{1}^{2}\right)\;\text{and}\\ \Delta \;y=y_{i}-y_{j}=G\left(0,2\sigma_{2}^{2}\right) \end{array}$$ (3.3)

where *G*(*μ*, *σ*^2^) denotes a Gaussian RV with mean *μ* and variance *σ*^2^. The joint pdf of the differences is given by

$$p_{\Delta x,\Delta y}\left(\alpha ,\beta \right)=\frac{1}{4\pi \sigma_{1}\sigma_{2}}\exp\left\{-\frac{1}{2}\left[\frac{\alpha^{2}}{2\sigma_{1}^{2}}+\frac{\beta^{2}}{2\sigma_{2}^{2}}\right]\right\}.$$ (3.4)

The cumulative probability distribution function for the distance between two mobiles is formulated in terms of the squares of the Gaussian RVs Δ*x* and Δ*y* as

$$F_{d}\left(\gamma \right)=\mathrm{Pr}\left\{d_{ij}\leq \gamma \right\}=\mathrm{Pr}\left\{\sqrt{{\left(\Delta x\right)}^{2}+{\left(\Delta y\right)}^{2}}\leq \gamma \right\}.$$ (3.5a)

Let us define the rectangular-to-polar change of variables given by Δ*x* = Δ*d_ij_* cos *θ* and Δ*y* = *d_ij_* sin *θ*. The joint pdf of *d_ij_* and *θ*, expressed in terms of the dummy variables *ρ* and *ϕ*, is found to be

$$\begin{array}{c} p_{d,\theta }\left(\rho ,\phi \right)=\frac{\rho }{4\pi \sigma_{1}\sigma_{2}}\exp\left\{-\frac{\rho^{2}}{4}\left[\frac{\cos^{2}\phi }{\sigma_{1}^{2}}+\frac{\sin^{2}\phi }{\sigma_{2}^{2}}\right]\right\},\\ 0\leq \phi \leq 2\pi ,\;\rho \geq 0. \end{array}$$ (3.6a)

The marginal pdf of *d_ij_* is found by integrating out the variable *ϕ* in Eq. (3.6a). Noting that the joint density is the same in each of the four quadrants, we can write

$$\begin{array}{l} p_{d}\left(\rho \right)=4\int_{0}^{\pi /2}d\phi \;p_{d,\theta }\left(\rho ,\phi \right)\\ \;=\frac{\rho }{\pi \sigma_{1}\sigma_{2}}\int_{0}^{\pi /2}d\phi \exp\left\{-\frac{\rho^{2}}{4}\left[\frac{\cos^{2}\phi }{\sigma_{1}^{2}}+\frac{\sin^{2}\phi }{\sigma_{2}^{2}}\right]\right\}\\ \;=\frac{\rho }{\pi \sigma_{1}\sigma_{2}}\int_{0}^{\pi /2}d\phi \exp\left\{-\rho^{2}\left(a+b\;\cos\;2\phi \right)\right\}\\ \;=\frac{\rho }{2\pi \sigma_{1}\sigma_{2}}\int_{0}^{\pi }d\alpha \exp\left\{-\rho^{2}\left(a+b\;\cos\;\alpha \right)\right\} \end{array}$$ (3.6b)

$$=\frac{\rho }{2\sigma_{1}\sigma_{2}}e^{-a\rho^{2}}I_{0}\left(b\rho^{2}\right)$$ (3.6c)

in which we use the integral in Ref. [5], Sec. 9.6.16 to identify I_0_(·), the modified Bessel function of the first kind, and we define

$$a≜\frac{1}{8}\left(\frac{1}{\sigma_{1}^{2}}+\frac{1}{\sigma_{2}^{2}}\right),\;b≜\frac{1}{8}\left(\frac{1}{\sigma_{1}^{2}}-\frac{1}{\sigma_{2}^{2}}\right).$$ (3.6d)

For convenience of notation and ease of comparison of the rectangular and Gaussian deployment models, we define the normalized variable *ξ*≜*ρ*/*D*_1_ = *ρ*/*κ*σ_1_, where *κ*≜*D_i_*/σ*_i_* relates the dimensions of the rectangular deployment area to the standard deviation of the Gaussian deployment distribution, and we denote the area shape parameter by *ζ* = *D*_1_/*D*_2_ = *σ*_1_/*σ*_2_ to be consistent with the use of this symbol for the rectangular deployment area. Then the pdf of the link distance can be written

$$\begin{array}{l} \;p_{d}(\rho =\kappa \sigma_{1}\xi )=\frac{1}{\kappa \sigma_{1}}\\ .\frac{\kappa^{2}\zeta \xi }{2}e^{-\kappa^{2}\xi^{2}(1+\zeta^{2})/8}I_{0}\left(\kappa^{2}\xi^{2}(1-\zeta^{2})/8\right),\;\rho \geq 0 \end{array}$$ (3.7a)

with the special case for ζ = 1 (*σ*_1_= *σ*_2_) given by

$$p_{d}\left(\rho =\kappa \sigma_{1}\xi \right)=\frac{1}{\kappa \sigma_{1}}.\frac{\kappa^{2}\xi }{2}e-\kappa^{2}\xi^{2}/4,\zeta =1,\rho \geq 0.$$ (3.7b)

**Fig. 7:**
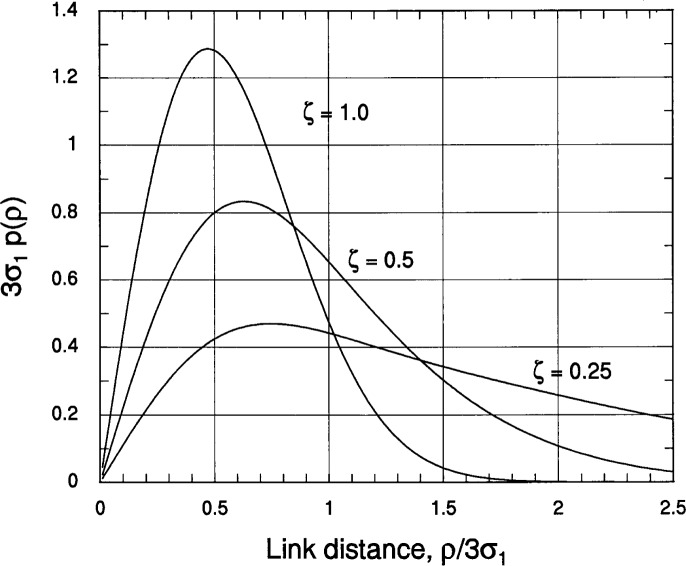
Plot of the link distance pdf for Gaussian-distributed mobile locations.

Plots of the link distance pdf Eq. (3.7a) are shown in Fig. 7 for *κ* = 3 (the length of the side of the rectangular deployment area is three times the standard deviation of the Gaussian deployment area in each direction) and *ζ* = 1, 0.5, and 0.25. The similarity of these plots to the in Fig. 5 is strong; the similarity can be made even stronger by choosing a little smaller value than *κ* = *D_i_*/σ*_i_* = 3. Of course, the curves in Fig. 7 are smoother than those in Fig. 5 because the deployment area for the assumption of a Gaussian distribution of mobile locations has no edges.

Now having the pdf of *d_ij_*, we can write the cdf Eq. (3.5a) for this RV as

$$\begin{array}{l} \;F_{d}\left(\gamma =\kappa \sigma_{1}\xi \right)=\int_{0}^{\gamma }d\rho \;p_{d}\left(\rho \right)\\ \;=\frac{1}{2\sigma_{1}\sigma_{2}}\int_{0}^{\gamma }d\rho \;\rho \;e^{-a\rho^{2}}I_{0}\left(b\rho^{2}\right)\\ =\frac{\kappa^{2}\zeta }{2}\int_{0}^{\xi }du\;u\;e^{-\kappa^{2}u^{2}\left(1+\zeta^{2}\right)/8}I_{0}\left(\kappa^{2}u^{2}\left(1-\zeta^{2}\right)/8\right). \end{array}$$ (3.8a)

For the special case of *ζ* = 1, Eq. (3.8a) becomes

$$\begin{array}{c} F_{d}(\gamma =\kappa \sigma_{1}\xi )=\frac{\kappa^{2}}{2}\int_{0}^{\xi }du\;u\;e^{-\kappa^{2}u^{2}/4}=\int_{0}^{\kappa^{2}\xi^{2}/4}dv\;e^{-v}\\ =1-e^{-\kappa 2\xi^{2}/4.} \end{array}$$ (3.8b)

**Fig. 8:**
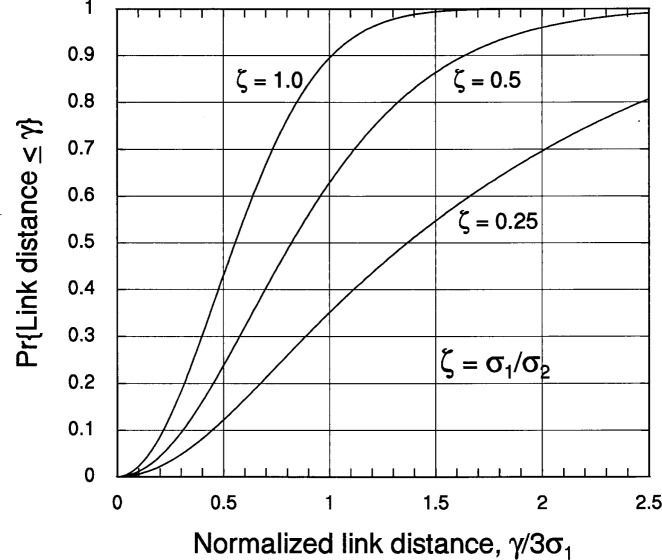
Plot of the link distance cdf for Gaussian-distributed mobile locations.

Plots of Eq. (3.8a) for *κ* = 3, obtained by numerical integration, are shown in Fig. 8.

### 3.2 Median and Mode for the Link Distance and Gaussian-Distributed Locations

For *ζ* = 1, Eq. (3.7b) is easily differentiated to find the mode of the distribution and Eq. (3.8b) is easily solved for the median:

$$\xi_{\mathrm{mode}}{|}^{\zeta =1}=\sqrt{2}/\kappa =1.4142,$$ (3.9a)

$$\xi_{\mathrm{med}}{|}^{\zeta =1}=2\sqrt{\text{ln}\;2}/\kappa =1.3863/\kappa .$$ (3.9b)

From Table 2, the mode of the distribution for a random distribution of mobile locations in a rectangular area for *ζ* = 1 is 0.4786; the mode for the Gaussian distribution of mobile locations for *ζ* = 1 matches it when *κ* = 2.9549. From Table 1, the median of the distribution for a random distribution of mobile locations in a rectangular area for *ζ* = 1 is 0.5120; the median for the Gaussian distribution of mobile locations for *ζ* = 1 matches it when *κ* = 2.7076.

## 4. Conclusions

We have found the distributions for the distance between randomly distributed mobiles for two different assumptions: (1) the mobile locations are uniformly distributed in a rectangular area, and (2) the mobile locations have a two-dimensional Gaussian distribution. The cdfs for both cases are very similar despite the fact that the first distribution has a finite boundary and the second does not. The implication of this finding is that for simulation or analysis of mobile communication systems, the model used for the distribution of the mobile locations can be chosen for convenience.

## Figures and Tables

**Table 1:** Median values of link distances for a *D*_1_ × *D*_2_ rectangular area, normalized by *D*_1_ < *D*_2_

*ζ* = *D*_1_/*D*_2_	ξ_med = med_/*D*_1_
1.00	0.5120
0.95	0.5254
0.90	0.5401
0.85	0.5563
0.80	0.5743
0.75	0.5943
0.70	0.6170
0.65	0.6428
0.60	0.6725
0.55	0.7072
0.50	0.7486
0.45	0.7990
0.40	0.8625
0.35	0.9465
0.30	1.0666
0.25	1.2453

**Table 2:** Mode of the link distances for a *D*_1_ × *D*_2_ rectangular area, normalized by *D*_1_ < *D*_2_

*ζ* = *D*_1_/*D*_2_	$\xi_{\mathrm{mode}}=\frac{\gamma_{\mathrm{mode}}}{D_{1}}$
1.00	0.4786
0.95	0.4908
0.90	0.5034
0.85	0.5165
0.80	0.5299
0.75	0.5439
0.70	0.5582
0.65	0.5730
0.60	0.5882
0.55	0.6037
0.50	0.6196
0.45	0.6357
0.40	0.6521
0.35	0.6687
0.30	0.6855
0.25	0.7023

## 5. Appendix A. Details of the Derivation of the Link Distance Distribution for a Rectangular Area

The development here follows that in [2] but in more detail, correcting several typographical errors in that presentation.

### 5.1 Evaluation for the Interval 0 ≤ *γ D*_1_, or 0 ≤ *ξ* ≤ 1

For this interval we use Eq. (2.6c); the upper limit of the first (outer) integral equals *ξ* and the upper limit of the second (inner) integral equals $\zeta \sqrt{\xi^{2}-u^{2}}$. Then,

$$\begin{array}{l} F_{d}\left(\gamma =\xi D_{1}\right)=4\int_{0}^{\xi }du\left(1-u\right)\int_{0}^{\zeta \sqrt{\xi^{2}-u^{2}}}dv\left(1-v\right)\\ =4\xi \int_{0}^{1}dw\left(1-\xi w\right)\int_{0}^{\zeta \xi \sqrt{1-w^{2}}}dv\left(1-v\right)\\ =2\xi \int_{0}^{1}dw\left(1-\xi w\right)\left[1-{\left(1-\zeta \xi \sqrt{1-w^{2}}\right)}^{2}\right]\\ =2\xi \int_{0}^{1}dw(1-\xi w)[2\zeta \xi \sqrt{1-w^{2}}-\zeta^{2}\xi^{2}(1-w^{2})]\\ =4\zeta \xi^{2}\int_{0}^{1}dw\sqrt{1-w^{2}}-4\zeta \xi^{3}\int_{0}^{1}dw\;w\sqrt{1-w^{2}}\\ \;-2\zeta^{2}\xi^{3}\int_{0}^{1}dw\left(1-\xi w-w^{2}+\xi w^{3}\right). \end{array}$$ (5.1a)

From Ref. [3], Sec. 3.251.1 we have

$$\int_{0}^{1}dw\;w^{u-1}{\left(1-w^{\lambda }\right)}^{\nu -1}=\frac{1}{\lambda }B\left(\frac{u}{\lambda },\nu \right)$$ (5.1b)

where B(*a*, *b*) = Γ(*a*) Γ(*b*)/Γ(*a* + *b*) is the Beta function. Applying Eq. (5.1b) to (5.1a) yields

$$\begin{array}{l} F_{d}\left(\gamma =\xi D_{1}\right)=4\zeta \xi^{2}\cdot \frac{1}{2}B\left(\frac{1}{2},\frac{3}{2}\right)-4\zeta \xi^{3}\cdot \frac{1}{2}B\left(1,\frac{3}{2}\right)\\ \;-2\zeta^{2}\xi^{3}\left(1-\frac{1}{2}\xi -\frac{1}{3}+\frac{1}{4}\xi \right)\\ \;=\zeta \xi^{2}\left[\pi -\frac{4}{3}\xi \left(1+\zeta \right)+\frac{1}{2}\zeta \xi^{2}\right]. \end{array}$$ (5.1c)

### 5.2 Evaluation for the Interval *D*_1_ *≤ γ ≤ D*_2_, or 1 ≤ *ξ* ≤1/ζ

For this interval we use Eq. (2.6c); the upper limit of the first (outer) integral equals 1 and the upper limit of the second (inner) integral equals $\zeta \sqrt{\xi^{2}-u^{2}}$. Then,

$$\begin{array}{l} F_{d}\left(\gamma =\xi D_{1}\right)=4\int_{0}^{1}du\left(1-u\right)\int_{0}^{\zeta \sqrt{\xi^{2}-u^{2}}}dv\left(1-v\right)\\ =2\int_{0}^{1}du\left(1-u\right)\left[1-{\left(1-\zeta \sqrt{\xi^{2}-u^{2}}\right)}^{2}\right]\\ =2\int_{0}^{1}du\left(1-u\right)\left[2\zeta \sqrt{\xi^{2}-u^{2}}-\zeta^{2}\left(\xi^{2}-u^{2}\right)\right]\\ =4\zeta \int_{0}^{1}du\sqrt{\xi^{2}-u^{2}}-4\zeta \int_{0}^{1}du\;u\sqrt{\xi^{2}-u^{2}}\\ \;-2\zeta^{2}\int_{0}^{1}du\left(\xi^{2}-\xi^{2}u-u^{2}+u^{3}\right). \end{array}$$ (5.2a)

From Ref. [4], integral No. 157 we have

$$\int du\sqrt{\xi^{2}-u^{2}}=\frac{1}{2}\left[u\sqrt{\xi^{2}-u^{2}}+\xi^{2}\sin^{-1}\left(\frac{u}{\xi }\right)\right]$$ (5.2b)

and in Ref. [4], integral No. 162 we have

$$\int du\;u\sqrt{\xi^{2}-u^{2}}=-\frac{1}{3}{\left(\xi^{2}-u^{2}\right)}^{3/2}.$$ (5.2c)

Substituting Eqs. (5.2b) and (5.2c) in Eq. (5.2a), we obtain

$$\begin{array}{c} F_{d}\left(\gamma =\xi D_{1}\right)=2\zeta \left[\sqrt{\xi^{2}-1}+\xi^{2}\sin^{-1}\left(\frac{1}{\xi }\right)\right]\\ -\frac{4}{3}\zeta \left[\xi^{3}-{\left(\xi^{2}-1\right)}^{3/2}\right]-\zeta^{2}\left(\xi^{2}-\frac{1}{6}\right)\\ =\frac{2}{3}\zeta \sqrt{\xi^{2}-1}\left(2\xi^{2}+1\right)+2\zeta \xi^{2}\sin^{-1}\left(\frac{1}{\xi }\right)\\ -\frac{1}{6}\zeta \left[8\xi^{3}+6\zeta \xi^{2}-\zeta \right]. \end{array}$$ (5.2d)

### 5.3 Evaluation for the Interval $D_{2}\leq \gamma \leq \sqrt{D_{1}^{2}+D_{2}^{2}}$, or $\zeta^{-1}\leq \xi \leq \sqrt{1+\zeta^{-2}}$

For this third interval we use Eq. (2.7c); the lower limit of the first (outer) integral equals $\sqrt{\xi^{2}-\zeta^{-2}}$and the lower limit of the second (inner) integral equals $\zeta \sqrt{\xi^{2}-u^{-2}}$. Then,

$$F_{d}(\gamma =\xi D_{1})=1-4\int_{\sqrt{\xi^{2}-\zeta^{-2}}}^{1}du(1-u)\int_{\zeta \sqrt{\xi^{2}-u^{-2}}}^{1}dv(1-v)$$ (5.3a)

$$\begin{array}{l} =1-2\int_{\sqrt{\xi^{2}-\zeta^{-2}}}^{1}du(1-u){\left(1-\zeta \sqrt{\xi^{2}-u^{2}}\right)}^{2}\\ =1-2\int_{\sqrt{\xi^{2}-\zeta^{-2}}}^{1}du(1-u)\left[1-2\zeta \sqrt{\xi^{2}-u^{2}}+\zeta^{2}\left(\xi^{2}-u^{2}\right)\right]\\ =1-2\int_{\sqrt{\xi^{2}-\zeta^{-2}}}^{1}du(1-u)+4\zeta \int_{\sqrt{\xi^{2}-\zeta^{-2}}}^{1}du\sqrt{\xi^{2}-u^{2}}-4\zeta \int_{\sqrt{\xi^{2}-\zeta^{-2}}}^{1}du\;u\sqrt{\xi^{2}-u^{2}}-2\zeta^{2}\int_{\sqrt{\xi^{2}-\zeta^{-2}}}^{1}du(\xi^{2}-\xi^{2}u-u^{2}+u^{3})\\ =1-{\left(1-\sqrt{\xi^{2}-\zeta^{-2}}\right)}^{2}+2\zeta \left[\sqrt{\xi^{2}-1}+\xi^{2}\sin^{-1}\left(\frac{1}{\xi }\right)-\zeta^{-1}\sqrt{\xi^{2}-\zeta^{-2}}-\xi^{2}\sin^{-1}\left(\frac{\sqrt{\xi^{2}-\zeta^{-2}}}{\xi }\right)\right]+\frac{4}{3}\zeta \left[{\left(\xi^{2}-1\right)}^{3/2}-\zeta^{-3}\right]-2\zeta^{2}\left[\xi^{2}-\frac{1}{2}\xi^{2}-\frac{1}{3}+\frac{1}{4}-\xi^{2}\sqrt{\xi^{2}-\zeta^{-2}}++\frac{1}{2}\xi^{2}(\xi^{2}-\zeta^{-2})\frac{1}{3}{\left(\xi^{2}-\zeta^{-2}\right)}^{3/2}-\frac{1}{4}{\left(\xi^{2}-\zeta^{-2}\right)}^{2}\right]\\ =\frac{2}{3}\zeta \sqrt{\xi^{2}-1}(2\xi^{2}+1)+2\zeta \xi^{2}\left\{\sin^{-1}\left(\frac{1}{\xi }\right)-\cos^{-1}\left(\frac{1}{\zeta \xi }\right)\right\}+\frac{2}{3}\sqrt{\xi^{2}-\zeta^{-2}}(2\zeta^{2}\xi^{2}+1)-\frac{1}{2}\zeta^{2}(\xi^{4}+2\xi^{2}-\frac{1}{3})+\frac{1}{6}\zeta^{-2}-\xi^{2}. \end{array}$$ (5.3b)

### 5.4 Special Case: *D*_1_ = *D*_2_ or ζ = 1

When *D*_1_ = *D*_2_ = *D* or ζ = 1, the interval from ξ = 1 to ξ = ζ^−1^ vanishes, and from Eqs. (5.1c) and (5.3b) the cumulative probability distribution for the distance between any two mobiles becomes Eq. (5.4). A form of the result for this special case was published in [1].

$$F_{d}(\gamma =\xi D)=\{\begin{array}{rr} 0, & \xi <0\\ \xi^{2}(\frac{1}{2}\xi^{2}-\frac{8}{3}\xi +\pi ), & 0\leq \xi <1\\ \frac{4}{3}\sqrt{\xi^{2}-1}(2\xi^{2}+1)-(\frac{1}{2}\xi^{4}+2\xi^{2}-\frac{1}{3}) & \\ +2\xi^{2}[\sin^{-1}(1/\xi )-\cos^{-1}(1/\xi ), & 1\leq \xi <\sqrt{2}\\ 1, & \xi \geq \sqrt{2} \end{array}.$$ (5.4)
